# Role of Computed Tomography and Other Non-Invasive and Invasive Imaging Modalities in Cardiac Allograft Vasculopathy

**DOI:** 10.3390/jcdd12070249

**Published:** 2025-06-27

**Authors:** Siddhant Passey, Jagriti Jha, Nirav Patel, Vincent Lipari, Saurabh Joshi, Raymond McKay, Joseph Radojevic, Joseph Ingrassia

**Affiliations:** 1 Department of Internal Medicine, University of Connecticut School of Medicine, Farmington, CT 06030, USA; passey@uchc.edu; 2 Department of Internal Medicine, Hartford Hospital, Hartford, CT 06106, USA; 3 Department of Pediatrics, University of Connecticut School of Medicine, Farmington, CT 06030, USA; 4 Department of Cardiology, Hartford Healthcare Heart and Vascular Institute, Hartford Hospital, Hartford, CT 06106, USAvincent.lipari@hhchealth.org (V.L.); saurabh.joshi@hhchealth.org (S.J.); raymond.mckay@hhchealth.org (R.M.);

**Keywords:** coronary allograft vasculopathy, invasive coronary angiography, coronary computed tomography angiography, nuclear imaging, intravascular imaging

## Abstract

Cardiac allograft vasculopathy (CAV) is a leading cause of allograft dysfunction and failure. CAV prevention, early detection, and management are essential to increasing allograft survival. In this comprehensive review, we discuss various invasive and non-invasive modalities that are being utilized for CAV detection. Invasive coronary angiography provides a visualization of vascular anatomy but is limited in detecting the microvasculature and diffuse and early structural changes. The addition of intracoronary assessment techniques, including intravascular ultrasound, optical coherence tomography, and coronary flow reserve assessment, offer(s) superior sensitivity in identifying CAV. Non-invasive imaging modalities, such as cardiac magnetic resonance imaging, computed tomography angiography, and positron emission tomography, provide complementary insights into CAV with myocardial perfusion and allograft function while reducing procedural risks. Our aim is to guide clinicians in selecting appropriate imaging strategies tailored to individual recipients, to improve detection, monitoring, and outcomes in CAV.

## 1. Introduction

Cardiac allograft vasculopathy (CAV) remains a leading cause of morbidity and mortality following heart transplantation (HTx). The prevalence of CAV is ~29% at five years and ~50% at 10 years following HTx despite improvement in immunosuppressive regimen and stricter comorbidity management [1,2,3]. The prognosis following diagnosis of CAV within three years is significantly worse with a five-year survival rate ~76% compared to ~82% without CAV [2].

CAV is a multifaceted arteriopathy leading to diffuse concentric intimal thickening of coronary vessels including microvascular segments [4]. The diagnosis of CAV is often delayed as HTx recipients do not develop angina due to complete denervation, and they typically present with non-specific symptoms of heart failure, arrhythmia, syncope, or sudden death in the late stages of CAV [5]. Therefore, routine surveillance is the cornerstone of early diagnosis of CAV prior to any clinical sequela. Invasive Coronary Angiogram (ICA) remains the gold standard for the diagnosis and grading of CAV [6], but its role in detecting early stages and diffuse and microvascular involvement is limited. Additionally, there is potential procedural risk and concern for contrast-induced nephropathy which can be prohibitive in some recipients [5].

Several non-invasive imaging modalities provide safer diagnostic options compared to an invasive approach. These include single-photon emission computed tomography (SPECT), positron emission tomography (PET), and coronary computed tomography angiography (CCTA). In recent years, PET myocardial perfusion imaging (MPI) has been promising in its sensitivity and accuracy, especially with microvascular involvement [7].

In this review, we compare various diagnostic invasive and non-invasive modalities that are being utilized for CAV. The relative strengths, limitations, and clinical applicability of each modality are discussed here to improve CAV-related outcomes in HTx recipients.

## 2. Pathophysiology of Cardiac Allograft Vasculopathy

CAV is a complex disease involving interactions between immune and non-immune factors that result in diffuse neointimal hyperplasia with a concentric remodeling of both the epicardial and microvascular coronary circulation [8]. Immune mechanisms occur with the activation of endothelial cells leading to presentation of donor human leukocyte antigens (HLA) class I and II [9], while a mismatch of HLA epitopes between the donor and recipient leads to the activation of cell-mediated and humoral immune systems. Activated T lymphocytes release pro-inflammatory cytokines, such as interleukins and tumor necrosis factors, which leads to the recruitment of monocytes and macrophages to the site. These macrophages further release cytokines and growth factors causing endothelial inflammation, smooth muscle proliferation, fibrosis, and extracellular matrix enlargement leading to vascular remodeling that can result in protrusion into the lumen with diffuse narrowing [5]. The role of humoral immunity is evidenced by circulating antibodies against the donor HLA which further amplifies the inflammatory response in the endothelium, contributing to CAV development [10]. The proliferation signal inhibitors Sirolimus and Everolimus have shown efficacy in slowing CAV by impeding lymphocyte proliferation and preventing the multiplication of fibroblasts and smooth muscle cells [11]. Further, induction with Rabbit-Thymoglobulin has been associated with decreasing CAV, which is likely accomplished through its mechanism of T cell depletion [12]. On the other hand, a study on de novo transplant recipients that were treated with rituximab with a goal to reduce CAV, showed an unexpected rise in CAV as detected by intravascular ultrasound (IVUS) compared to controls, indicating that disruption of normal B cell activity by CD20 antagonism may be harmful [13] and a need for further research into the immune-mediated mechanisms of CAV.

Other non-immune mechanisms such as diabetes, dyslipidemia, older age, male sex, obesity, ischemia–reperfusion injury during transplantation, and viral infection may lead to vascular injury and remodeling resulting in CAV [9]. Insulin resistance and dyslipidemia are commonly observed in 50–80% of transplant receipts and further contribute to endothelial dysfunction and atherogenesis [14]. High-sensitivity C-reactive protein is often found to be elevated with a level of ~4.1 mg/L in those with progressive CAV compared to ~1.8 mg/L in those without CAV, also correlating with disease severity [15]. Thus, CAV results from the interplay between local vascular inflammation and systemic inflammatory responses. Cumulatively, these immunological and non-immunological mechanisms act concurrently, leading to chronic endothelial dysfunction and interruption in nitric oxide production, providing an environment in which this unique vasculopathy has a tendency toward rapid progression.

## 3. Invasive Coronary Angiography

**Figure 1:**
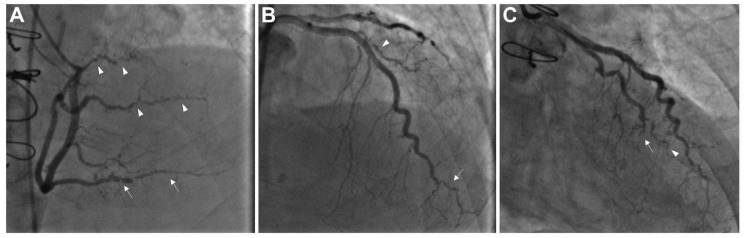
Coronary angiography illustrating advanced cardiac allograft vasculopathy (CAV), classified as ISHLT CAV3. (**A**) The right coronary artery shows severe stenosis and distal pruning in the posterior descending artery (indicated by arrows) and the posterolateral branches (arrowheads). (**B**) The distal portion of the left anterior descending artery and its diagonal branch exhibit severe disease and distal pruning (arrow and arrowhead, respectively). (**C**) The distal left circumflex artery with chronic total occlusion (arrow) and the obtuse marginal branch displays marked distal pruning (arrowhead). CAV—cardiac allograft vasculopathy; ISHLT—International Society for Heart and Lung Transplantation. Adapted from Shahandeh et al., 2022 [22].

ICA is the gold standard modality for CAV diagnosis and grading severity according to the recommendations of International Society for Heart and Lung Transplantation (ISHLT). The frequency of ICA is not well established and varies among various transplant programs. In clinical practice, it is typically being performed early following HTx and then annually or biannually based on risk factors and renal function [6]. The recent ISHLT guidelines have graded CAV severity into four classes (Table 1): CAV0 (absent), CAV1 (mild), CAV2 (moderate), and CAV3 (severe), based on arterial stenosis and graft dysfunction (Figure 1) [16]. The risk of graft failure is higher with higher CAV grading. In comparison to patients without CAV, the hazard ratio for death or retransplantation in patients with CAV3 was ~5.71 compared to ~1.22 for CAV1 [17].

ICA provides direct visualization of coronary anatomy with high temporal resolution as well as spatial resolution and is useful in identifying significant focal luminal stenosis, which can also be addressed during the procedure. Additionally, ICA is a widely available and clinically accepted modality. Angiographic evidence of CAV is present in approximately 10–20% of HTx recipients within the first year, increasing to about 30% by the fifth year, and close to 50% by the tenth year [17,18]. Recipients with angiographic evidence of CAV have a 19% risk of progressing to severe disease within five years, and with severe CAV, there is a 50% likelihood of death or the need for retransplantation within five years, underscoring its prognostic utility [19].

ICA, however, has limitations as it only assesses the arterial lumen and not the endothelial wall, leading to less sensitivity in detecting diffuse or early stages of CAV [20]. In a histopathological analysis of CAV, 73% of coronary segments were reported normal by angiography despite significant intimal hyperplasia, therefore potentially missing the diagnosis [21]. The use of ICA is further constrained by potential vascular complications, radiation, and the possibility of contrast-induced nephropathy. ICA combined with intracoronary assessment improves sensitivity in the detection of CAV.

## 4. Intravascular Imaging Modalities

The limitations of ICA in detecting CAV have been addressed with the addition of advanced imaging modalities, such as IVUS and optical coherence tomography (OCT), which provide complementary high-resolution wall characterization and detection of early, usually concentric, intimal thickening.

With respect to IVUS, a prior 1995 study by Tuzcu et al. demonstrated a sensitivity of 42% and a specificity of 95% of coronary angiography compared to IVUS for detecting CAV [23]. In a 1992 study by St Goar et al., 70% of patients had a normal angiogram, but all of these recipients had at least minimal (grade I) measurable thickness by IVUS at 1 year and beyond following HTx. Moreover, 50% of these patients had moderate (grade III, defined as intimal thickness 0.3–0.5 mm or >0.5 mm in <180-degree vessel circumference) to severe intimal thickening (grade IV, defined as intimal thickness >0.5 mm in >180-degree vessel circumference, or > 0.1 mm in any one area of vessel circumference) [24].

**Figure 2:**
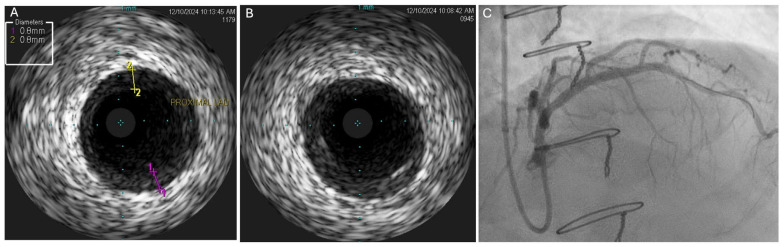
Discordance between findings of coronary angiography and IVUS. (**A**,**B**) IVUS of proximal LAD demonstrating mild concentric homogeneous intimal thickening in the proximal LAD with a maximal thickness of 0.8 mm, corresponding to severe intimal thickening (grade IV, as the thickening is greater than 0.5 mm in > 180 degrees of the vessel). (**C**) Coronary angiogram demonstrating very mild nonobstructive narrowing of the very proximal LAD which correlated with area of concentric homogenous thickening on IVUS. IVUS—intravascular ultrasound; LAD—left anterior descending artery.

Multivessel IVUS imaging enhances sensitivity, with a notable increase in the detection of CAV (Figure 2). This technique increased the prevalence of CAV from 39% with single-vessel imaging to 74% with three-vessel imaging at three years [25]. This contrast represents an advantage of multivessel imaging in detecting CAV at earlier stages, potentially leading to more effective monitoring and interventions. In addition to the early detection of CAV by IVUS, it also provides prognostication. Rapidly progressive vasculopathy, defined as an increase in minimal intimal thickness (MIT) by ≥0.5 mm within a year of HTx, has been associated with worse outcomes with rate of mortality or graft loss of 20.8% at 5 years compared to 5.9% (*p* = 0.007) in the patient group that had a maximal intimal thickness increase <0.5 mm at 1 year [26]. Another study by Tuzcu et al. showed that rapidly progressive MIT had higher rates of death and myocardial infarction [27].

It is, however, important to note that these studies provided data in the era when cyclosporine and azathioprine were standard immunosuppressive therapies as opposed to tacrolimus, mycophenolate, statins, and proliferation signal inhibitors, which are known to delay CAV progression and were not widely utilized [20,28,29]. These advances in post-transplant management pose questions regarding the applicability of historical IVUS parameters in contemporary practice. For example, it was recently demonstrated that there was no statistically significant association between major adverse cardiovascular events and rapidly progressive vasculopathy (0.5 mm MIT change at 1 year), which was theoretically attributed to the contemporary changes in management. Studies have also demonstrated newer IVUS parameters, such as MIT changes of ≥0.35 mm between 1 and 5 years to also be associated with major adverse cardiovascular events (MACE) and mortality [30]. While IVUS has attained primacy over coronary angiography for the diagnosis of early CAV [31], routine IVUS surveillance for CAV is not universally adopted due to the cost, procedural complexity, technical limitations, and increased anticoagulation requirement.

In short, IVUS remains a key modality in the early detection of CAV, giving important prognostic information and guiding therapy. Its integration with conventional angiography and other emerging technologies optimizes diagnostic yield and clinical outcomes. However, pragmatic considerations include cost, expertise, and evolving therapeutic regimens which call for ongoing refinement in its application in the modern era of transplant cardiology.

**Figure 3:**
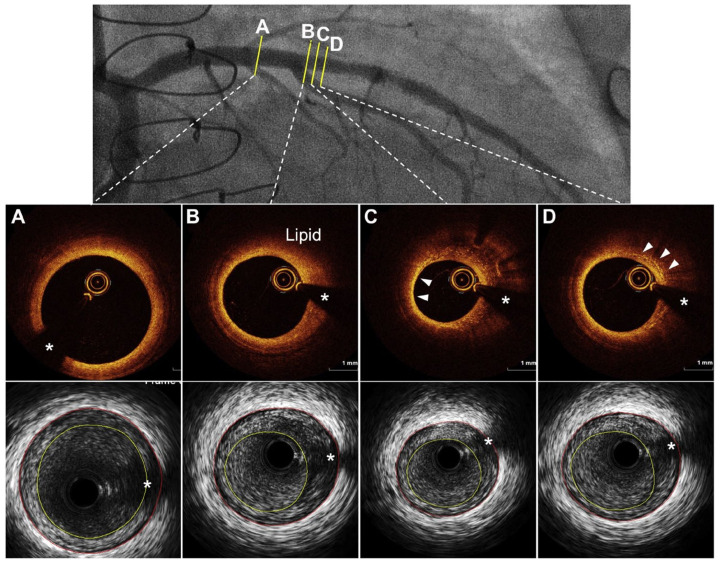
Cardiac allograft vasculopathy through intravascular imaging: optical coherence tomography and intravascular ultrasound demonstrating. (**A**) Concentric fibrous plaque, (**B**) lipid-rich plaque, (**C**) arrowheads showing thin-cap fibroatheroma, and (**D**) arrowheads showing accumulation of macrophages. * Guidewire artifact. Adapted from Shahandeh et al., 2022 [22].

OCT is an advanced imaging technique that utilizes near-infrared light to produce high-resolution cross-sectional images of the coronary arteries (Figure 3). It provides a spatial resolution approximately 10 times higher compared to IVUS [32,33]. OCT is more effective than both ICA and IVUS, as a high resolution allows for near-histologic visualization of coronary vessel layers enabling the precise measurement of intimal and medial thickness and detailed tissue characterization. OCT can classify plaques into one of three types, fibrous, calcific, or lipid-rich, each with characteristic features [34], and is uniquely sensitive in the detection of vulnerable plaques, including thin-cap fibroatheromas (fibrous caps < 65 μm thick), infiltration by macrophages, and microchannels [35]. Improved plaque characterization and reduced interobserver variability in measuring luminal dimensions and intimal thickness are advantages of OCT compared to IVUS.

There is a strong correlation between OCT and IVUS in the measurement of CAV, and both modalities had complete agreement with each other in one study, but both OCT and IVUS had higher sensitivity than ICA in identifying CAV by 72% and 47%, respectively [36]. Moreover, it was demonstrated that intimal thickness greater than 100 μm was detected by OCT in 67% of artery segments but by IVUS in only 14% of segments, showing higher sensitivity for very early intimal hyperplasia by OCT [37]. The OCT for the Characterization of Cardiac Allograft Vasculopathy after Heart Transplantation (OCTCAV) study proposed using an intima-to-media ratio greater than 1 as a threshold of abnormal intimal thickening, further supporting OCT as an important modality in early CAV detection [38].

However, OCT has limited tissue penetration and is therefore unable to assess deeper structures such as a lipid core or calcium deposits behind a thick fibrous cap [39]. The need for radiocontrast to flush the vessel represents a disadvantage in patients with kidney disease. Although further technological advancements, including combined OCT-IVUS catheters, may overcome many of the current limitations, they are not widely available yet. Although there are substantial sensitivity and mechanistic insights with OCT, its value for prognostication and prediction of long-term outcomes in HTx recipients remains underexplored. Longitudinal studies are needed to assess whether OCT adds incremental prognostic benefit.

## 5. Non-Invasive Imaging Modalities

### 5.1. Cardiac Magnetic Resonance Imaging (CMR)

**Figure 4:**
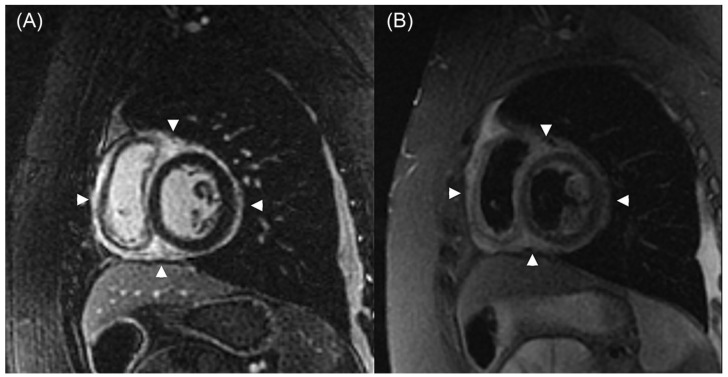
CMR shows a widespread epicardial distribution of LGE in the setting of graft dysfunction. In Panel (**A**), a short-axis, post-contrast inversion recovery gradient echo image at the midventricular level reveals diffuse epicardial enhancement in both ventricles (arrowheads). Panel (**B**) shows a corresponding short-axis T2-weighted triple inversion recovery fast spin-echo image, displaying increased signal intensity (arrowheads) in the same region, consistent with myocardial edema. Adapted from Anand et al., 2024 [47]. CMR—cardiac magnetic resonance imaging; LGE—late gadolinium enhancement.

**Figure 5:**
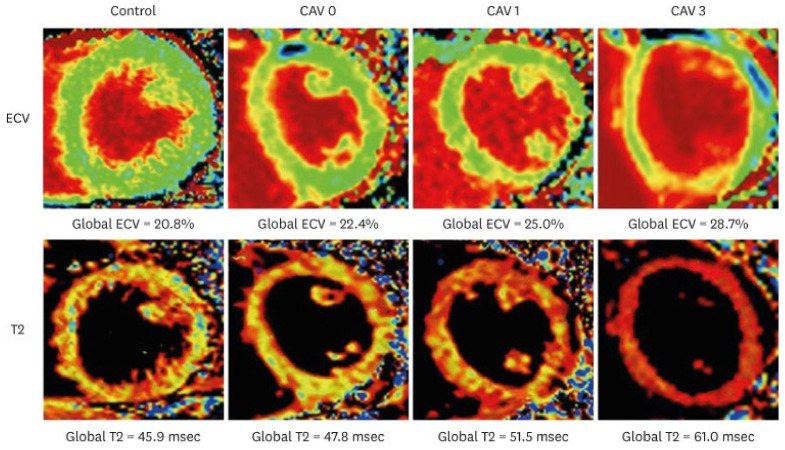
T2 and ECV maps in a healthy control and heart transplantation recipient representative of the different CAV subgroups. Adapted from Abbasi et al., 2022 [48].

CMR is a promising imaging modality for detecting myocardial perfusion abnormalities and fibrosis in cardiac allograft vasculopathy (CAV). Stress perfusion CMR measures myocardial perfusion reserve (MPR) as a marker of microvascular disease. In a cross-sectional study, an MPR ≤ 1.68 demonstrated 100% sensitivity and 100% negative predictive value (NPV) for CAV detection (IVUS—defined MIT >0.5 mm). These data suggest a role for CMR as a “rule-out” test for CAV; although, notably, the test had a limited specificity of 63% [40]. CMR is also effective in fibrosis assessment using late gadolinium enhancement (LGE) (Figure 4) and through an assessment of pre- and post-contrast T1-mapping for the quantification of extracellular volume [41,42]. Inflammation and edema may be assessed through T2 mapping (Figure 5). LGE infarct-type patterns correlate with CAV severity and have also been utilized to identify myocardial scarring, believed to be related to early CAV [43]. A study by Hughes et al. demonstrated that the prevalence of myocardial fibrosis increased with higher ISHLT CAV grades (Table 1), and that both the prevalence and extent of myocardial fibrosis were significantly associated with an increased risk of MACE [44]. While fibrosis may indicate advanced disease, CMR’s ability to detect subclinical changes supports its role in risk stratification and long-term monitoring. A study by Miller et al. demonstrated that CMR-derived MPR significantly outperformed ICA in detecting both moderate and severe CAV. Specifically, the area under the receiver operating characteristic curve (AUC) for CMR was 0.89 for moderate CAV and 0.88 for severe CAV, compared to 0.59 and 0.67 for coronary angiography, respectively [45]. The above studies led to the inclusion of CMR-derived MPR and LGE in the evaluation of CAV as a class IIB recommendation in ISHLT 2023 guidelines (Table 2) [6].

Additionally, CMR-derived MPR and diastolic strain metrics help identify early microvascular dysfunction and independently predict microvascular dysfunction on endomyocardial biopsy [46]. Oxygenation-sensitive CMR has also been explored, and patients without CAV have demonstrated impaired myocardial oxygenation, suggesting microvascular dysfunction. Patients with severe CAV have also shown to have an impaired oxygen-sensitive response compared to patients without CAV [42]. As a non-invasive, radiation-free technique, CMR offers significant advantages for comprehensive myocardial assessment, positioning it as a valuable tool for early CAV detection and prognosis in HTx recipients. Further studies should refine its role in clinical practice.

### 5.2. Cardiac Computed Tomography Angiography (CCTA)

**Figure 6:**
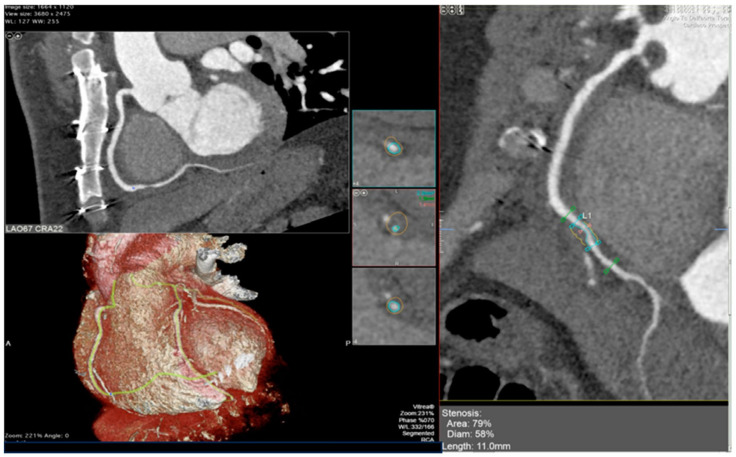
CCTA reveals grade 3 CAV based on ISHLT criteria. A calcified plaque is identified in the second segment of the right coronary artery, shown in the reconstructed view on the right panel and corresponding orthogonal views on the left panel. Adapted from Pergola et al., 2023 [62]. CCTA—Cardiac Computed Tomography Angiography; CAV—Cardiac allograft vasculopathy; ISHLT—International Society for Heart and Lung Transplantation.

CCTA is a non-invasive imaging modality assessing both the coronary arterial wall and lumen, making it valuable for CAV detection (Figure 6). A meta-analysis of 13 studies comparing CCTA with ICA found a sensitivity of 94%, specificity of 92%, and NPV of 99%, highlighting its reliability for detecting significant CAV (stenosis ≥50%). Benchmarked against IVUS, 64-slice CCTA showed 81% sensitivity and 75% specificity for detecting ≥0.5 mm intimal thickening [49]. The 2025 CCTA-HTx Study evaluated CCTA’s diagnostic performance for CAV in 37 HTx patients at 5 years compared to invasive methods. CCTA, incorporating qualitative and quantitative plaque analysis, and FFR assessment, identified CAV in 62.2% of patients, with its best diagnostic model achieving 84% accuracy, 83% sensitivity, and 86% specificity. Adding FFR improved sensitivity but reduced overall specificity and accuracy. CCTA also required less radiation than invasive approaches to achieve high-quality images using newer-generation scanners [50].

CCTA also evaluates coronary artery calcium (CAC), a marker of atherosclerosis. Studies have shown that CCTA can also help identify CAV through CAC. This is highlighted by a study that showed the absence of CAC on CT as having an NPV of 97% for ISHLT CAV2-3 and 88% for significant stenosis on ICA. CAC was also associated with poor outcomes in HTx patients in the long term [51]. Another CCTA-based technique is quantitative coronary plaque analysis, which utilizes semiautonomous software (such as Autoplaque 2.0; Cedars-Sinai Medical Center) to give absolute volumes for total plaque, calcified plaque, non-calcified plaque, fibrofatty plaque, and fibrous plaque. This technique allows for further improvement in the diagnostic performance in the detection CAV [52].

Recently, the fat attenuation index (FAI) measured from CCTA has drawn growing interest due to its potential to predict adverse cardiovascular outcomes in individuals with CAD disease who have not undergone heart transplantation [53]. Although large scale studies are not yet conducted in transplant patients, two cohort studies of 149 subjects and 39 transplant patients have been performed recently. Both studies showed higher baseline FAI values across major coronary arteries that were associated with increased risk of cardiac mortality or retransplantation. FAI demonstrated strong intra-patient and inter-vessel reproducibility, supporting its reliability in serial imaging. These findings indicate that FAI may enhance the utility of CCTA in non-invasive surveillance for the early detection of cardiac allograft vasculopathy [54,55].

CCTA not only images coronary arterial anatomy but also provides functional assessment on a non-invasive basis, even in the presence of increased heart rates. The fractional flow reserve calculated from CT (FFRct) can be retrospectively applied to images obtained in already-acquired CCTA datasets [56]. A recent long-term study included 106 patients sampled 6 to 13 years after HTx at the time of the baseline CCTA were included. The study showed that FFRct measurements for the distal segments of large coronary arteries decreased over two years, paralleling the rise in anatomical coronary stenoses [57]. These data indicate that following changes in the FFRct over time could provide an early indication of functional deterioration due to CAV, providing a novel non-invasive tool for ongoing post-transplant monitoring. Additional studies will be required to determine its role in clinical decision-making. The utility of physiological assessment of coronary flow in CAV will be discussed later in this review.

Despite its advantages, CCTA has limitations. Its spatial resolution restricts visualization of smaller vessels (<1.5 mm) [58]. Elevated heart rates in transplant recipients can also compromise image quality. This is due to the lower temporal resolution of CCTA compared to ICA, leading to motion artifacts that can obscure diagnostic details [59], though newer CT technologies such as dual-source CT (DSCT) and motion correction algorithms have improved accuracy [60]. Radiation exposure is another concern; additionally, CCTA requires contrast posing a risk for nephropathy, particularly in transplant patients with renal impairment [61]. Overall, CCTA is a promising non-invasive modality for CAV detection, offering high sensitivity, specificity, and NPV. The 2023 ISHLT guidelines have included a class IIa recommendation for CCTA as a non-invasive alternative to ICA in CAV detection (Table 2) [6]. However, its limitations, including lower temporal resolution, heart rate dependency, and contrast nephropathy risk, must be weighed when determining its suitability for individual patients.

### 5.3. Nuclear Imaging

SPECT and PET use radiotracers for MPI but differ remarkably in spatial resolution, quantification, and diagnostic accuracy. Among the most common MPI techniques, SPECT detects perfusion abnormalities after the induction of stress by exercise or pharmacologic agents. It uses radiotracers such as technetium-99 m (Tc) or thallium-201 (Tl) to visualize myocardial blood flow. Diagnostic accuracy depends on the stress agent used, the radiotracer, and the CAV criteria. In prior studies, pharmacologic stress testing using dobutamine SPECT in heart transplant patients has demonstrated 83–90% sensitivity and 55–87% specificity [63,64,65], while dipyridamole-stress SPECT showed 92% sensitivity and 86% specificity in one study [66]. A more recent large retrospective study included 503 at a median of 9 years post-transplant who underwent either exercise or pharmacological stress MPI at the physician’s discretion and found relatively modest accuracy of SPECT in diagnosing high-grade CAV (CAV2-3), with an AUC of 0.65 [67]. This modest performance is often attributed to widespread CAV in heart transplant recipients, which can result in balanced ischemia where relative perfusion defects may be missed.

**Figure 7:**
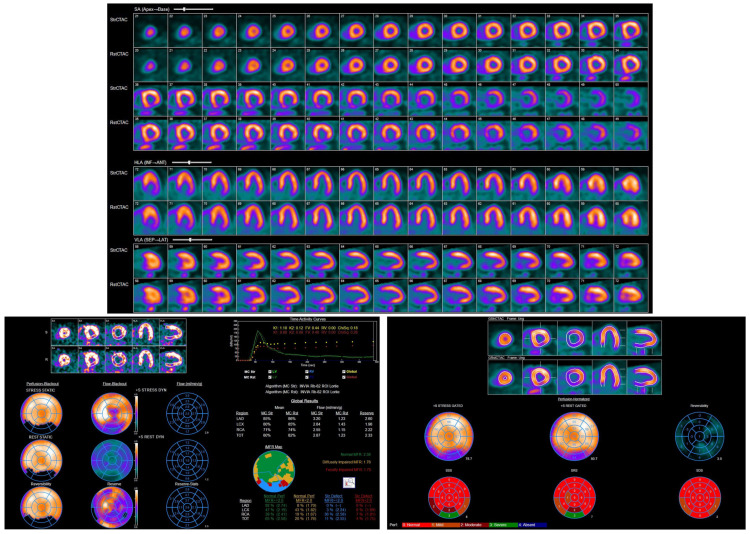
Rubidium-82 PET images demonstrating abnormal left ventricular perfusion. A medium-sized, moderate-intensity scar was noted in the mid-to-basal inferior and inferolateral myocardium, with no inducible ischemia.

On the other hand, PET has better spatial and temporal resolution which makes it a technique for the qualitative as well as quantitative assessment of myocardial perfusion. The fact that it can estimate absolute myocardial blood flow (MBF) and myocardial flow reserve (MFR) places it in an advantageous position regarding the detection of early, diffuse CAV that may be underestimated by SPECT due to balanced ischemia [68]. In a retrospective study of ammonia-13 PET for the detection of CAV2-3, it was seen that compared to the standard myocardial index alone (AUC 0.82), a multi-parametric scoring that also added left ventricular ejection fraction ≤45% and stress myocardial blood flow <1.7 improved the diagnostic value (AUC 0.88) [69]. Another prospective study by Mc Arlde et al. studied dipyridamole rubidium-82 PET and noted that a MFR ≤ 1.75 was associated with a fourfold increase in adverse cardiac events (HR 4.41, *p* = 0.006) and a sixfold increase in mortality (HR 6.4, *p* = 0.008) [70]. However, early post-transplant reductions in MBF tend to resolve within a year, making differentiation between transient endothelial dysfunction and progressive vascular disease important [71] (Figure 7).

There are other limitations to both modalities. SPECT is widely available, inexpensive, and is a well-established technique compared to PET but has the drawback of lower sensitivity in mild CAV by virtue of its semi-quantitative nature and the potential for balanced ischemia to result in false negatives [72]. The choice of the vasodilatory stress agent also affects SPECT safety as adenosine hypersensitivity in denervated hearts may post a risk of atrioventricular block, though regadenoson is shown to be safer [73]. On the other hand, PET gives better diagnostic performance, particularly in obese patients, as it minimizes attenuation artefacts [74]. However, it is more expensive, less accessible, and lacks universally accepted thresholds for the detection of CAV, thus needing further research for standardization. Despite these differences, both SPECT and PET fulfill complementary roles in CAV assessment. SPECT continues to represent a pragmatic approach for routine surveillance and excluding severe disease, whereas PET excels in the early detection of microvascular dysfunction, risk stratification, and prognostication. Further technological advancements with regard to hybrid imaging, specifically SPECT/CT and PET/CT, quantitative perfusion analysis, and new radiotracers hold promise for increasing their clinical value, further optimizing long-term outcomes in HTx recipients. For instance, a recent study demonstrates that ammonia-13 PET/CT myocardial perfusion imaging provides valuable prognostic information in HTx recipients, with PET CAV grade 0/1 showing high NPVs (0.93–0.95) for the development of severe CAV on three interval ICAs performed up to 1200 days. Compared to PET CAV 0, patients with PET CAV 2/3 had a significantly increased risk of all-cause mortality (HR 2.86, 95% CI 1.36–6.00, *p* = 0.006), and in a sensitivity analysis of subset of patients that had stable ISHLT CAV on consecutive ICA, CAV 2/3 was still associated with a higher likelihood of death or retransplantation (HR 3.20, 95% CI 1.18–8.69, *p* = 0.03) [75]. Another recent study demonstrates that PET MBF at one year post-transplant correlates with epicardial CAV supporting the role of PET for early non-invasive CAV assessment [76].

### 5.4. Echocardiography

Echocardiography is a non-invasive tool for assessing cardiac function in HTx recipients. Resting two-dimensional echocardiogram has limited accuracy in CAV detection [77], especially for mild cases. While wall motion abnormalities (WMA) may indicate disease, their absence does not rule it out [78]. Tissue Doppler Imaging (TDI) improves sensitivity, with a radial peak systolic velocity <10 cm/s linked to a 97% likelihood of CAV, while >11 cm/s virtually excludes severe disease with 90% probability [79]. Dobutamine stress echocardiography (DSE) is widely used for CAV surveillance due to its radiation-free, cost-effective, and validated nature. However, diagnostic performance varies: a meta-analysis (n = 749) reported a sensitivity of 60% and specificity of 86% [80]. In a large cohort of 497 patients undergoing surveillance DSE, only 1.8% of DSE studies were positive for ischemia, suggesting very low incidence of positive results on surveillance DSE [81]. Advancements like strain imaging and speckle tracking have improved DSE’s accuracy [82,83]. However, DSE struggles to detect early, non-flow-limiting CAV, making negative results unreliable. Despite its limitations, integrating strain imaging, speckle tracking, and CFR assessment may enhance echocardiography’s role in early CAV detection and risk stratification, improving outcomes in HTx patients.

The major strengths and limitations of some of the invasive and non-invasive imaging modalities are summarized in Table 3.

## 6. Emerging Hybrid Imaging

### 6.1. Near-Infrared Spectroscopy (NIRS)

Near-infrared spectroscopy (NIRS) has emerged as a promising intravascular imaging technique for evaluating CAV by detecting lipid-rich plaques within the coronary arteries. NIRS has been integrated with IVUS into a single catheter, allowing for simultaneous assessment of both plaque composition and arterial structure [84]. A combined OCT-NIRS imaging catheter has been developed as a proof-of-concept model [85]. A study by Zheng et al. compared CAV to native atherosclerosis using NIRS-IVUS. The study found that transplant patients displayed early and rapid lipid buildup with a lower plaque burden and lower calcium levels compared to patients with native atherosclerosis [86]. Another study studied NIRS-IVUS by combining IVUS-derived plaque burden (PB) and NIRS-derived lipid core burden index (LCBI). The PB + LCBI combined model significantly improved the accuracy for fibroatheroma detection [87]. While NIRS lacks depth resolution and long-term outcome data in CAV, its integration with IVUS enhances its adoption and potential prognostic value in heart transplant recipients.

### 6.2. Near-Infrared Autofluorescence (NIRAF)

Near-infrared autofluorescence (NIRAF) imaging is often combined with OCT to provide both structural and compositional information about the plaque. While OCT offers high-resolution images of the plaque’s microstructure, NIRAF allows for detection of plaque instability, as it detects high-risk morphological features in coronary plaques such as intraplaque hemorrhage and heme degradation products [88]. For instance, Ughi et al. showed that NIRAF intensity is elevated in plaques with high-risk phenotypes, such as fibroatheromas and plaque ruptures, which are relevant in the context of CAV [89]. Furthermore, Kunio et al. provided histopathological correlation of NIRAF in human cadaver coronary arteries, indicating that NIRAF is able to detect intraplaque hemorrhage and ceroid, both of which are markers of oxidative stress and rapid lesion progression [90]. This suggests that NIRAF can offer complementary information to traditional imaging modalities, potentially aiding in the early detection and management of CAV.

## 7. Physiological Assessment of Coronary Flow in CAV

Coronary flow reserve (CFR) and fractional flow reserve (FFR) have been extensively studied for their role in assessing coronary physiology in CAV. CFR obtained by intracoronary Doppler flow probes provides an approximation of microvascular integrity which has been proved to deteriorate progressively after the cardiac transplantation process. In an early study involving 29 HTx patients, Treasure et al. found that microvascular endothelium-dependent vasodilation, measured as flow reserve, decreased with time after transplantation, which was hypothesized to reflect CAV development [91]. In a subsequent study, patients with minimal epicardial disease also had lower CFR and an increased risk for ventricular dysfunction [92]. Tona et al. then showed that a CFR ≤ 2.5, measured at approximately four years post-transplantation, independently predicted the development of epicardial CAV and was associated with increased mortality, independent of angiographic findings [93].

Physiological assessment has been more recently complemented by the introduction of the measurement of the FFR, which is often obtained during invasive angiography. FFR measures pressure gradients across stenotic lesions and provides a functional assessment of whether myocardial blood flow is reduced [94]. Traditional FFR measurements conducted during invasive coronary angiography involve passing a pressure sensing guidewire and determining the ratio of pressure distal to the stenotic coronary lesion and the aortic pressure during induced hyperemia. The vessel FFR (vFFR), computed from computational fluid dynamics applied to three-dimensional quantitative coronary angiography, does not require invasive pressure wire insertion or pharmacologic hyperemia [95]. In two analyses by Fearon and colleagues, HTx patients undergoing both wire-based FFR and IVUS assessments exhibited striking results, even in the absence of significant angiographic stenosis. A subset of these patients exhibited an FFR ≤ 0.80, which strongly correlated with IVUS-derived plaque burden metrics, most notably plaque area and volume. In particular, patients with an FFR ≤ 0.80 demonstrated a mean plaque volume of about 37%, while those with an FFR > 0.80 had a plaque volume of about 23%. In contrast, FFR values presented a rather low correlation with focal stenosis indices [96,97]. More recently, it was shown that a traditional wire-based FFR ≤ 0.90 at early measurement following HTx was associated with increased late mortality or the need for retransplantation, although these findings require further validation [98].

Recent studies have also utilized vFFR as an effective modality in measuring CAV. In a study of heart transplant recipients, vFFR identified functional impairment in 32% of cases where standard angiographic classification did not detect disease [95]. Another study also reported an excellent correlation between vFFR and wire-based FFR [99]. The quantitative flow ratio (QFR) is another emerging alternative in the assessment of coronary physiology and is computed from angiographic and intravascular imaging data. In one study, a QFR threshold of 0.88 also had a significantly high sensitivity of 94% for the prediction of the future development of at least non-obstructive CAV [100]. However, these methods will require further validation before their wide clinical use. Overall, physiological assessment in combination with imaging techniques like ICA, IVUS, and OCT represents a more complete approach to CAV, offering early detection and risk stratification while overcoming the limitations of isolated physiological measurement.

## 8. Artificial Intelligence (AI) in CAV Imaging

Applications of AI imaging in CAV are actively under investigation. Machine learning algorithms in echocardiography have enabled the automatic evaluation of global longitudinal strain (GLS) as a sensitive marker of subclinical myocardial dysfunction. For instance, Salte et al. showed that a novel AI-based method could automatically measure GLS with high accuracy and minimal operator input, significantly reducing the time required for analysis and eliminating measurement variability [101]. Similarly, Stowell et al. validated an open-source machine-learning-based GLS methodology, which showed strong agreement with expert consensus measurements in terms of accuracy and reproducibility [102]. Deep learning techniques further extended diagnostic performance with CMR and CCTA. For instance, Pezel et al. developed a machine learning model that integrates both CMR and CCTA data to predict MACE in patients with newly diagnosed coronary artery disease (CAD). This model outperformed traditional risk scores and individual imaging modalities, achieving an area under the receiver operating characteristic curve (AUC) of 0.86, compared to 0.55–0.83 for other methods [103]. Moreover, Chen et al. demonstrated that a deep learning-based vascular extraction and stenosis detection technique applied to CCTA could accurately identify obstructive CAD with an AUC of 0.78, showing comparable performance to human readers but with significantly reduced analysis time [104]. Further AI model development specifically to HTx patients may provide personalized risk stratification and further refine surveillance algorithms through multimodal imaging dataset integration to predict CAV progression more accurately.

## 9. Future Directions

Despite the improvement in multimodal imaging, various gaps still remain in optimizing CAV detection and management. The adoption of newer technologies is dependent upon outcome-driven validation studies and further endorsement by clinical guidelines. Finally, standardized protocols that integrate both invasive and non-invasive modalities are warranted to improve reproducibility and clinical utility. Further research into AI-driven diagnostic models and hybrid imaging platforms will likely shape the future of CAV monitoring, paving the way for a more personalized and data-driven approach to post-transplant care.

## 10. Conclusions

In summary, CAV is a major cause of mortality in heart transplant patients and often does not cause angina due to complete denervation in transplant recipients. Early detection of CAV is essential to prevent clinical complications. A variety of invasive and non-invasive imaging modalities are utilized in detecting CAV. ICA remains the only class I recommendation in the most recent ISHLT 2023 guidelines and should be considered yearly or every other year for evaluation. Intravascular imaging modalities such as IVUS and OCT are now recommended as class IIa recommendations and have further enhanced the ability to detect intimal thickening for an earlier diagnosis of CAV; their high resolution also allows for better vessel wall assessment and the detection of plaque composition. Physiological assessment of CFR allows for the detection of small-vessel CAD.

Non-invasive imaging modalities offer a relatively safer option for CAV detection. CCTA is recommended as a class IIa recommendation and offers high negative predictive value but is limited in detecting smaller vessels due to elevated resting heart rates in transplant recipients. PET MPI is also a class IIa recommendation and can estimate MBF and MFR allowing for the qualitative and quantitative assessment of myocardial perfusion. CMR is a class IIb recommendation and allows for allows for the detection of MPR as a marker of microvascular disease, with high sensitivity and NPV. SPECT and DSE are also class IIb recommendations in adults and offer limited sensitivity compared to other modalities. Further research is needed to determine standardized protocols integrating both invasive and non-invasive modalities and to determine the role of AI-driven diagnostic models in CAV monitoring.

## Figures and Tables

**Table 1:** International Society for Heart and Lung Transplantation (ISHLT) grading of cardiac allograft vasculopathy (CAV).

ISHLT CAV Grade	Angiographic Findings
CAV 0	No angiographic coronary artery lesions detected
CAV 1 (Mild)	Angiographic stenosis <50% in any left main coronary artery or <70% stenosis in primary vessel or branch
CAV 2 (Moderate)	Angiographic stenosis <50% in the left main coronary artery with stenosis ≥70% in a single primary vessel or stenosis ≥70% in isolated branches across two different systems.
CAV 3 (Severe)	Angiographic stenosis ≥50% in left main coronary artery, or stenosis ≥70% in ≥2 primary vessels, or ≥70% isolated branch stenosis in all 3 systems; also includes patients with evidence of allograft dysfunction or evidence of restrictive physiology

Primary Vessel: proximal and middle one-third of the LAD, LCX, ramus, and dominant or co-dominant RCA. Secondary branch vessel: distal one-third of the primary vessels or any segment within a large septal perforator, diagonal or obtuse marginal branches, or any section of a non-dominant RCA. LAD: Left anterior descending artery; LCX: Left circumflex artery; RCA: Right coronary artery.

**Table 2:** International Society for Heart and Lung Transplantation (ISHLT) 2023 recommendations for coronary allograft vasculopathy (CAV) surveillance post-transplant.

Imaging Modality	ISHLT 2023 Recommendations for CAV Surveillance	Class of Recommendation	Level of Evidence
Invasive Coronary Angiography (ICA)	ICA is recommended for evaluating the progression of CAV, with angiographic findings classified based on the 2010 ISHLT nomenclature.	Class I	B
	ICA should be considered yearly or every other year for evaluation. In patients with a higher risk of complications (eg. kidney disease), less frequent assessments may be appropriate.	Class I	C
Intravascular Ultrasound (IVUS)	Baseline IVUS, along with ICA, should be considered at 4 to 6 weeks post-HTx and again at 1 year post-HTx.	Class IIa	B
	In pediatric recipients, baseline IVUS along with IVUS may be considered at 1 year post-HTx.	Class IIb	C
Optical Coherence Tomography (OCT)	OCT, alongside ICA, may be considered at 4 to 6 weeks and 1 year post-HT.	Class IIa	B
Cardiac MRI (CMR)	CMR myocardial perfusion reserve and delayed gadolinium enhancement evaluation may be considered.	Class IIb	C
Cardiac CT Angiography (CCTA)	CCTA can serve as a non-invasive alternative to ICA for detecting CAV in epicardial vessels ≥2 mm.	Class IIa	B
PET Myocardial Perfusion Imaging (MPI)	PET MPI and myocardial blood flow quantification can be utilized for the non-invasive detection of CAV and for providing prognostic insights.	Class IIa	B
SPECT MPI	SPECT MPI has low sensitivity for detecting CAV but may be valuable for prognostication in HTx recipients who are unable to undergo invasive testing, CCTA, or PET.	Adult: Class IIb, Pediatrics: Class IIa	B
Dobutamine Stress Echocardiography (DSE)	DSE has low sensitivity for detecting CAV but may be valuable for prognostication in HTx recipients who are unable to undergo invasive testing, CCTA, or PET.	Adult: Class IIb, Pediatrics: Class IIa	B
Physiological Assessment	Assessment of CFR; IMR alongside ICA may be helpful in detecting small vessel CAD, a manifestation of CAV.	Class IIa	B

CAV: Coronary allograft vasculopathy; CFR: Coronary flow reserve; IMR: Index of microcirculatory resistance; CAD: Coronary artery disease; HTx: Heart transplant.

**Table 3:** Major strengths and limitations of invasive and non-invasive imaging modalities for the evaluation of CAV.

Imaging Modality	Strengths	Limitations
Coronary Angiography	Gold standard; widely available; potential for direct revascularization	Invasive, limited in detecting microvascular disease; procedural risks
Intravascular Ultrasound	Early detection of intimal thickening; high resolution for vessel wall assessment	Invasive; limited availability; higher cost; more expertise
Optical Coherence Tomography	High-resolution imaging of plaque composition; better detection of early CAV	Invasive; limited penetration depth; requires contrast; expensive; more expertise
Cardiac MRI	Non-invasive; no radiation exposure; detects myocardial fibrosis and microvascular disease; differentiation of infarcted and viable myocardium	Limited availability; contraindicated in some patients with metal implants; risk of nephrogenic systemic sclerosis in individuals with renal disease when gadolinium is used; motion artifacts
Cardiac Computed Tomography Angiography	Non-invasive; evaluates arterial wall and lumen; high negative predictive value	Limited availability; radiation exposure; contrast nephropathy risk; limited in small vessels; motion artifacts due to high resting heart rates
Positron Emission Tomography Myocardial Perfusion Imaging	Non-invasive; quantifies microvascular dysfunction; good prognostic value	Expensive; limited availability; radiation exposure
Single-Photon Emission Computed Tomography	Widely available; good specificity	Lower sensitivity; risk of false negatives due to balanced ischemia; requires clinical validation
Dobutamine Stress Echocardiography	Cost-effective; no radiation	Limited sensitivity; poor detection of early CAV

CAV: Coronary Allograft Vasculopathy.

## Data Availability

No new data were created or analyzed in this study. Data sharing is not applicable to this article.
